# Supplementary material for: Hollow Microcavity Electrode for Enhancing Light Extraction
Source: Micromachines (Basel). 2024 Feb 27;15(3):328. doi: 10.3390/mi15030328 (PMC10972122; doi:10.3390/mi15030328)
Supplement: Supplementary file 1 [file micromachines-15-00328-s001.zip › micromachines-2879770-supplementary.pdf]

Supplementary Materials

Hollow Microcavity Electrode for Enhancing Light Extraction

Seonghyeon Park [1,†], Byeongwoo Kang [1,†], Seungwon Lee [1], Jian Cheng Bi [1], Jaewon Park [1], Young Hyun Hwang [1], Jun-Young Park [1], Ha Hwang [1], Young Wook Park [2,*] and Byeong-Kwon Ju [1,*]

[1] Display and Nanosensor Laboratory, Department of Electrical Engineering, Korea University, Seoul 02841, Republic of Korea; gohnd@korea.ac.kr (S.P.); kang7369@korea.ac.kr (B.K.); lswon96@korea.ac.kr (S.L.); vlfrkatjd@korea.ac.kr (J.C.B.); pkjaewon@korea.ac.kr (J.P.); aksyp@korea.ac.kr (Y.H.H.); mrjoon123@korea.ac.kr (J.-Y.P.); ha08.hwang@samsung.com (H.H.)

[2] Department of Semiconductor and Display Engineering, Sun Moon University, Asan 31460, Republic of Korea

* Correspondence: zerook@sunmoon.ac.kr (Y.W.P.); bkju@korea.ac.kr (B.-K.J.)

† These authors contributed equally to this work.

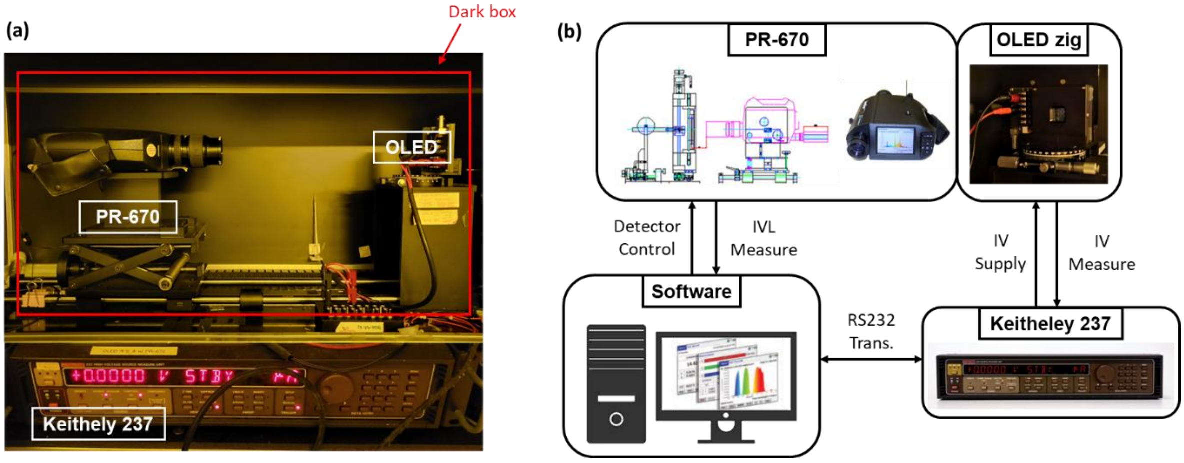

Figure S1. Equipment setup for OLED measurements. (a) Photo of the equipment. (b) Schematic diagram of measurement.

(a) (b) (c)

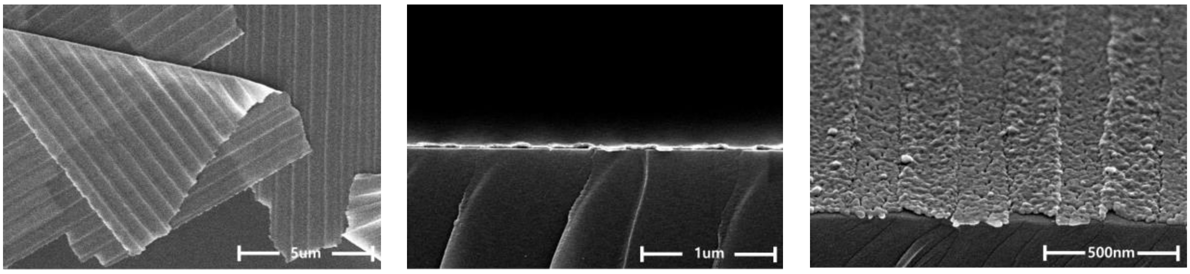

Figure S2. SEM images of fabricated Ag mirror-stripped photoresist without IZO. (a) The appearance of peeled Ag film. (b) The side view of aggregated Ag. (c) The top view of aggregated Ag.

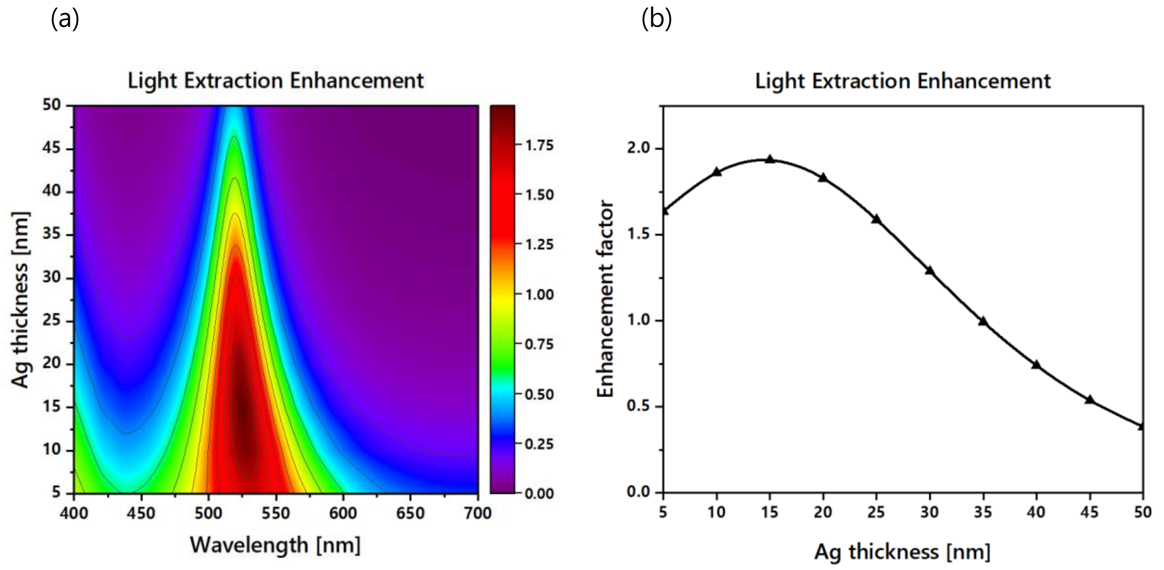

Figure S3. Optimization of IZO thickness. (a) Contour plot of the enhancement factor as the thickness of the Ag sweep depending on the wavelength. (b) Optimization of the pitch at a 525 nm wavelength.

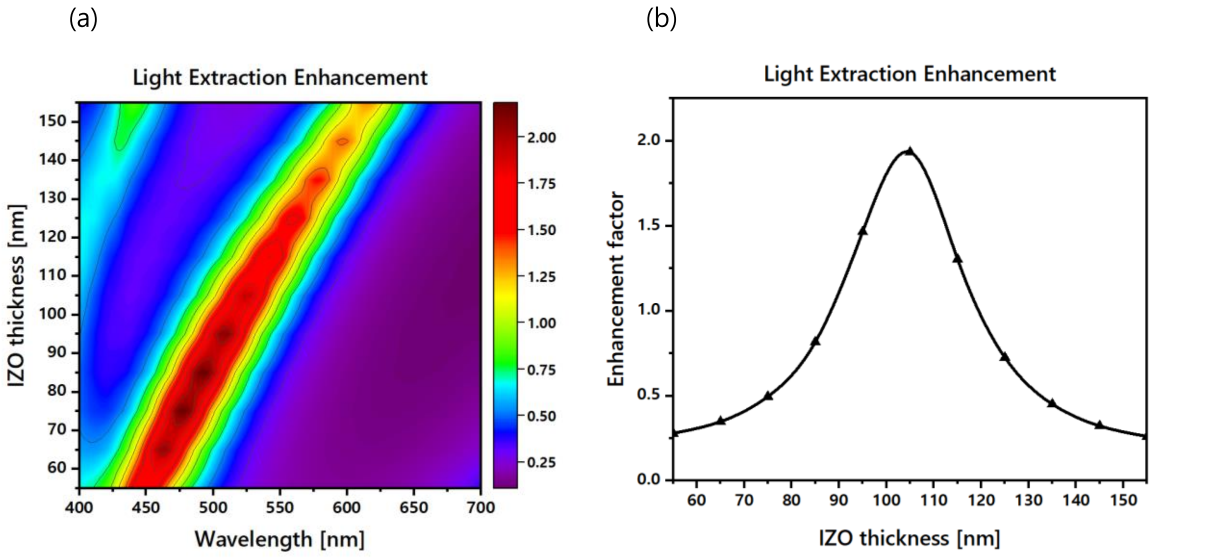

Figure S4. Optimization of IZO thickness. (a) Contour plot of the enhancement factor as the thickness of the IZO sweep depending on wavelength. (b) Optimization of the pitch at a 525 nm wavelength.

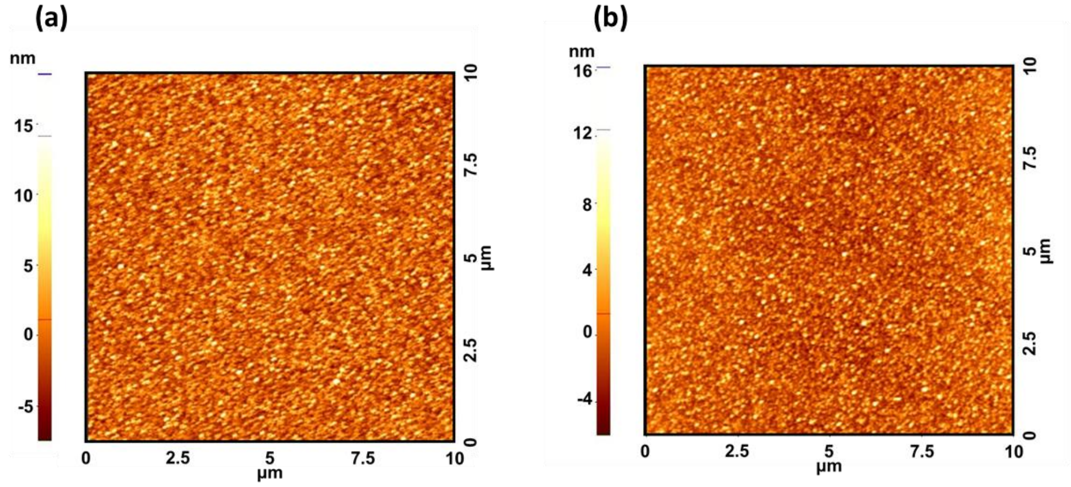

Figure S5. AFM images of the (a) Ag and (b) Ag/IZO structures.

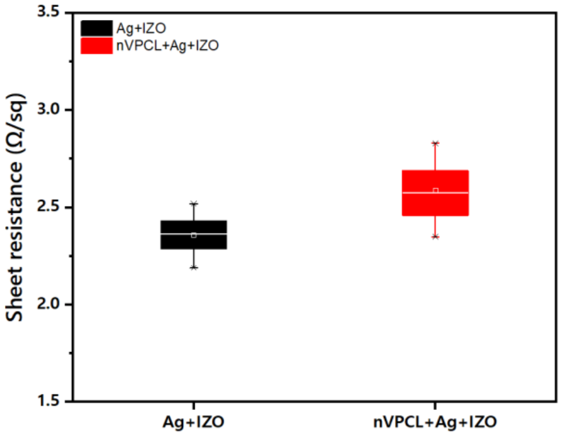

Figure S6. Sheet resistance of the Ag/IZO and nVPCL/Ag/IZO structures.

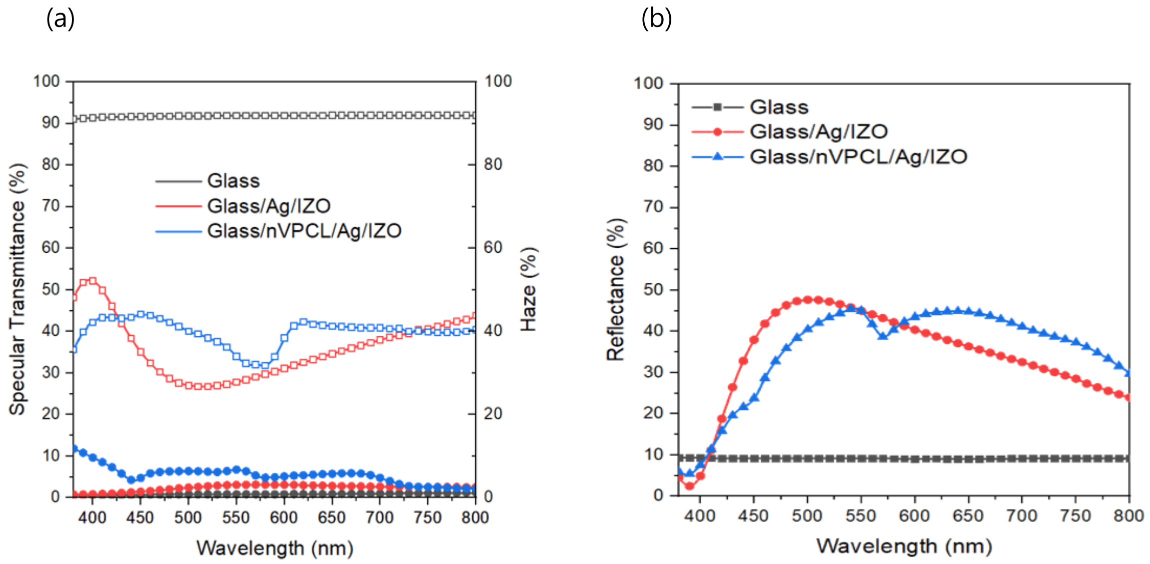

Figure S7. (a) Spectral transmittance and haze of fabricated electrodes. (b) Reflectance of fabricated electrodes.

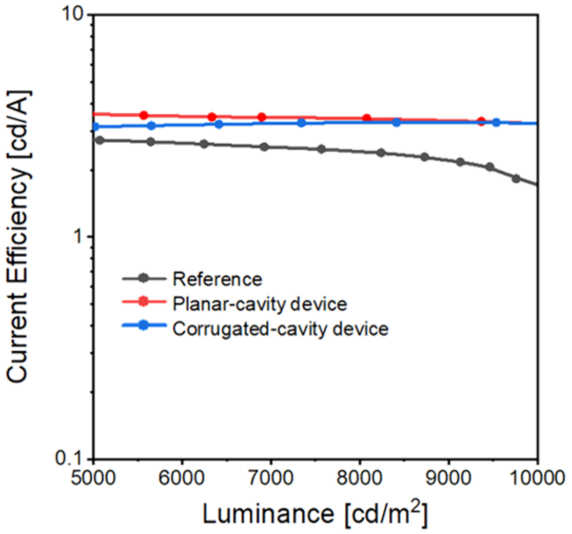

Figure S8. Current efficiency of the fabricated device.
